# Overexpression of LINC00672 promotes autophagy in Alzheimer’s disease by upregulating GPNMB

**DOI:** 10.1371/journal.pone.0322708

**Published:** 2025-05-14

**Authors:** Lingyi Gao, Shijun Hu, Yan Lv, Guoxian Zheng, Zhichuan Lin

**Affiliations:** 1 Department of Emergency, Hainan General Hospital, Haikou, China; 2 Department of Neurology, Hainan General Hospital, Haikou, China; Manipal Academy of Higher Education, INDIA

## Abstract

**Background:**

Alzheimer’s disease (AD) is an irreversible neurodegenerative brain disorder, and autophagy crafts a new dawn on AD therapeutics. However, whether LINC00672 exerts its biological effects involvement in autophagy-mediated mechanisms in AD remain obscure.

**Methods:**

SH-SY5Y cells were treated with Amyloid Beta 1–42 (Aβ1-42, Aβ), while an AD mouse model was established using streptozotocin (STZ). The effects of LINC00672 overexpression on cell proliferation, apoptosis, and autophagy were evaluated in Aβ-stimulated SH-SY5Y cells. Besides, the impact of LINC00672 on cognitive function and pathological changes of the hippocampal tissues were validated in AD mice. Additionally, the interaction between LINC00672 overexpression and GPNMB silencing were determined in vitro.

**Results:**

Aβ stimulation diminished viability, augmented apoptosis, restricted the activation of autophagy in SH-SY5Y cells, while these alterations were partially abolished by LINC00672 overexpression. Furthermore, LINC00672 upregulation could improve cognitive impairment, and attenuate neuronal damage and even death in the STZ-treated AD mice. Additionally, GPNMB knockdown aggravated the improved neuronal injury and relatively restrained autophagy in Aβ-stimulated cells after LINC00672 overexpression.

**Conclusions:**

LINC00672 exerted a protective effect in the AD progression by upregulating GPNMB to promote autophagy.

## Introduction

Alzheimer’s disease (AD) is an irreversible age-related neurodegenerative brain disorder that can lead to progressive cognitive decline and memory loss [1]. The prevalence of AD nowadays reaches near 50 million people and stands as roughly 60–70% of all dementia cases internationally [2,3]. Currently, cholinesterase inhibitors, NMDA-receptor antagonists, or their combination therapies are deemed as approved approaches in management of patients with AD, while these available therapies mainly focus on temporary symptomatic relief and there is no effective disease-modifying treatment to date at present [4,5]. Therefore, continued researches are of paramount importance to comprehend the aetiology characterization of AD, which contributes to developing more additional efficient therapies for AD.

Autophagy, an intracellular homeostatic process controls the elimination of damaged cell constituents, allowing their degradation and recycling into their fundamental components to generate cellular energy [6]. Recently, autophagy crafts a new possible dawn in the realm of neurodegenerative illnesses therapeutics opportunities [7]. For instance, Suppressor of Cytokine Signaling 2 downregulation improves autophagic dysfunction in Huntington’s disease [8]. Impaired autophagy in microglia is found to exacerbate dopaminergic neurodegeneration in Parkinson’s disease (PD) experimental model [9]. Furthermore, long noncoding RNA (lncRNA) RMRP augments autophagy-mediated neuronal apoptosis in AD process [10]. Therefore, the endorsement of autophagy serves as a promising novel therapeutic strategy in management of against AD, and in-depth researches on linked molecular mechanisms are still necessary.

LncRNAs serve as the preeminent mediators in brain development [11,12]. Furthermore, dysregulation of these lncRNAs is also linked to the suppression or stimulation of autophagy in diverse neurodegenerative illnesses [13]. LncRNA NEAT1 downregulation increases miR-374c-5p expression, and retards apoptosis and autophagy in PD mice [14]. Knockdown of lncRNA BACE1-AS ameliorates neuronal injury in AD, which is mediated by modulating autophagy via the miR-214-3p/ autophagy related gene (ATG)-5 axis [15]. Additionally, it is implicated that LINC00672 is participated in the process of AD [16]. However, the specific role of LINC00672 in modulation of autophagy and relevant mechanisms on AD remains to be investigated.

Glycoprotein Non-Metastatic Melanoma Protein B (GPNMB), a transmembrane glycoprotein, has recently emerged as a critical player in neurodegenerative diseases [17]. Studies demonstrate that GPNMB ameliorates cerebral edema and neuroinflammation, protects the blood-brain barrier, and enhances neurological function through modulation of the AMPK/NF-κB signaling pathway [18]. Elevated plasma levels of GPNMB in PD patients are linked to its interaction with α-synuclein, which exacerbates PD pathogenesis by promoting the uptake of pathological α-synuclein aggregates [19]. Furthermore, GPNMB serves as a promising biomarker for neuroinflammation in AD [20]. Notably, overexpression of GPNMB in AD mouse models enhances autophagic clearance of beta-amyloid, thereby attenuating AD pathology [21]. Collectively, these findings underscore the pivotal role of GPNMB in regulating autophagy and influencing the progression of neurodegenerative disorders, particularly AD. In this study, we attempted to unravel the specific regulatory effects of LINC00672 and GPNMB in AD through establishing in vitro and in vivo AD experimental models, as well as further to elaborate the possible involvement in autophagy-mediated interaction mechanisms. Findings of this investigation may open up the possibilities of therapeutic direction against AD.

## Materials and methods

### Cell culture and transfection

Human neuroblastoma cell line SH-SY5Y were procured from iCell (Shanghai, China), then cultivated in Dulbecco’s Modified Eagle Medium (Hyclone, Logan, UT, USA) with the presence of 10% fetal bovine serum (Hyclone), 100 U/mL penicillin and 100 μg/mL streptomycin (Hyclone) in a humidified incubator comprising 5% CO_2_ under 37°C. The cells were initially exposed to Amyloid Beta 1–42 (Aβ1-42, Aβ, Sigma-Aldrich Inc., St. Louis, MO, USA) at 0, 5, 10, 20 μM for 24 h, then the cells were treated with Aβ at 10 μM for 0, 12, 24, 48 h. Finally, the condition under 10 μM for duration period of 24 h was adopted for treating cells to construct the AD cell model.

For overexpressing LINC00672 or silencing GPNMB in SH-SY5Y cells, the cells were planted in 6-well plates until reaching approximately 70%-80% confluency per well. Subsequently, the LINC00672-overexpressed plasmid pcDNA3.1-LINC00672 (OE-SNCA), pcDNA-NC (OE-NC), siRNA-GPNMB and siRNA-NC synthesized by RiboBio Co., Ltd. (Guangzhou, China) were transfected into SH-SY5Y cells using Lipofectamine 3000 (Invitrogen, Carlsbad, CA, USA). The sequence information targeting GPNMB were presented in S1 Table.

### Animal models

Healthy male C57BL/6 mice (6–8 weeks old, 18–20 g) were procured from SPF Biotechnology Co., Ltd. (Beijing, China). All mice had unrestricted access to standard rodent chow and water and were housed in a 12:12 h light/dark cycle, with environmental conditions maintained at 20 ± 2°C and 40–60% humidity. Twenty-four mice were assigned to 4 groups randomly (n = 6 per group) including Control, AD (model), AD+OE-NC, and AD+OE-LINC00672. AD mouse models were constructed according to previously described methods; the streptozotocin (STZ, 3 mg/kg, Sigma) was slowly injected into the lateral ventricle of mice for 21 consecutive days [22]. The healthy mice in the Control group were injected with equivalent amount of sodium citrate buffer in the same location. For the overexpression group, the STZ-treated mice received stereotactic injection of LINC00672 lentivirus or negative controls (1 × 10^9^ IU/mL, 3 μL) into the CA1 area of the hippocampus [23]. In each group, the rats were tested by water maze to evaluate cognitive function. After lentivirus injection of 3 weeks, all the mice (no mice died) euthanized through inhalation of isoflurane (Rayward, Shenzhen, China) for 2–3 min. After that, the CA1 area of the hippocampus tissues were harvested for subsequent experiments. All animal experiments were approved by the Hainan General Hospital ethical committee (2022–209).

### Morris water maze test

Morris water maze experiment was applied to evaluate the memory and learning ability of mice. Briefly, a circular pool was divided into four equal areas, and a platform is placed in the center. The mice were placed in a random spot, and then allowed to search the hidden platform. Four training probe trials with a maximum of 120 s to find the safe platform were daily carried out for consecutive 7 days. After 24 hours of the last training trial, the test was conducted and a tracking system was used to monitor the escape latency (time to reach the platform) and the frequency of crossing the platform.

### Quantitative real‐time polymerase chain reaction (qRT‐PCR)

Total RNA extraction was implemented from cells or tissues using Trizol reagent (Invitrogen, Carlsbad, CA, USA). Thereafter, the PrimeScript RT Reagent kit (TaKaRa, Otsu, Japan) was utilized for cDNA synthesis. Subsequently, qRT-PCR analysis was conducted for detection of mRNA expression levels on the ABI7500 quantitative PCR instrument (Thermo Fisher Scientific, Waltham, MA, USA). The conditions were: 95°C for 30 s, 40 cycles of 95°C for 10 s and 60°C for 30 s. GAPDH was functioned as the internal reference by using an optimized comparative 2^−ΔΔCt^ technique, and the sequences of primers was provided in S1 Table.

### Western blot analysis

Total protein isolation and concentration determination were carried out using radioimmunoprecipitation assay lysis buffer (Beyotime, Shanghai, China) and BCA Protein Assay Kit (Beyotime), respectively. After that, the lysis products were run on sodium dodecyl sulfate polyacrylamide gel, and which was then shifted onto polyvinylidene fluoride membranes. After being occluded with 5% nonfat milk, the membranes were crossbred with primary antibodies against Beclin-1 (ab207612, 1:1000, Abcam, Cambridge, UK), LC3B (ab48394, 1:1000, Abcam), P62 (ab109012, 1:1000, Abcam), Atg4 (ab108322, 1:1000, Abcam), GPNMB (ab227695, 1:1000, Abcam), and GAPDH (5174, 1:1000, Cell Signaling Technology, Danvers, MA, USA) at 4°C overnight, and maintained in goat anti-rabbit secondary antibodies (1/5000, Abcam) the next day. Finally, the development of immunoblotting was determined utilizing imager system (Bio-Rad, Hercules, CA, USA), and gray values were quantified through ImageJ software (V1.8.0.112, NIH, Madison, WI, USA) with GAPDH as an endogenous control.

### Cell counting kit-8 (CCK-8) method

Briefly, the cells (1 × 10^4^ cells/well) were inoculated in a 96-well plate, and preincubated in complete medium at 37°C. After cultivating for 24 h, each well was full of 10 μL CCK-8 reagent (Beyotime) for additional 2 h exposure in incubators. Finally, the optical density of each well under the wavelength of 450 nm was recorded with a microplate reader (VL0000D0, Thermo Fisher Scientific).

### Cell apoptosis detection

The cells (5 × 10^5^ cells/well) were seeded in 6-well plates at 37°C for 24 h. Thereafter, the cells were collected, centrifugated, and resuspended in binding buffer, then subjected to AnnexinV-FITC (5 µL, Beyotime) and of propidium iodide (10 µL, Beyotime) for 15 min in the dark. Finally, the proportion of apoptotic cells were quantified using flow cytometry (BD Biosciences, San Jose, CA, USA).

### Reactive oxygen species (ROS) production

The cell samples after indicated treatment were reacted with 25 μM 2′,7′-dichlorodihydrofluorescein diacetate (DCFH-DA, Sigma) in the dark at 37°C for 30 min. Following washing, the fluorescence intensity was evaluated using fluorescence microscope (Leica Microsystems GmbH, Wetzlar, Germany).

### Hematoxylin and eosin (HE) staining

Isolated CA1 area of the hippocampus tissues were perfused with 4% paraformaldehyde, embedded in paraffin, and then sliced from the paraffin blocks at the 4 μm thickness. The slices underwent deparaffinage with xylene and rehydration with gradient concentrations of ethanol prior to staining using hematoxylin and eosin. Finally, the stained sections were fixed with neutral gum, and then photographed under the microscope (IX71, Olympus, Tokyo, Japan).

### Terminal deoxynucleotidyl transferase dUTP Nick End Labeling (TUNEL) assay

TUNEL staining was used for detecting neuronal apoptosis in CA1 area of the hippocampus tissues. Paraformaldehyde-fixed, paraffin-embedded tissue sections were de-paraffinized with xylene, dehydrated with gradient ethanol, rinsed with PBS, and kept in the protease K working solution. Subsequently, the slides were immersed in the 3% hydrogen peroxide, then coped with TUNEL assay solution (Beyotime). Finally, the samples were exposed to 4′,6-diamidino-2-phenylindole solution, and visualized with a fluorescence microscope (Leica Microsystems GmbH).

### Statistical analysis

The experimental data compiled from at least 3 independent replicates were analyzed by GraphPad Prism 9.0 (GraphPad Software Inc., San Diego, CA, USA) software with reported as mean ± standard deviation. When comparisons between two groups were implemented, the student’s t-test was employed. The One-Way Analysis of Variance in combination with Tukey’s test was applied to compare the mean of multiple groups. A *p* value less than 0.05 represented statistical significance.

## Results

### Overexpression of LINC00672 ameliorates Aβ-treated damage in SH-SY5Y cells

**Fig 1:**
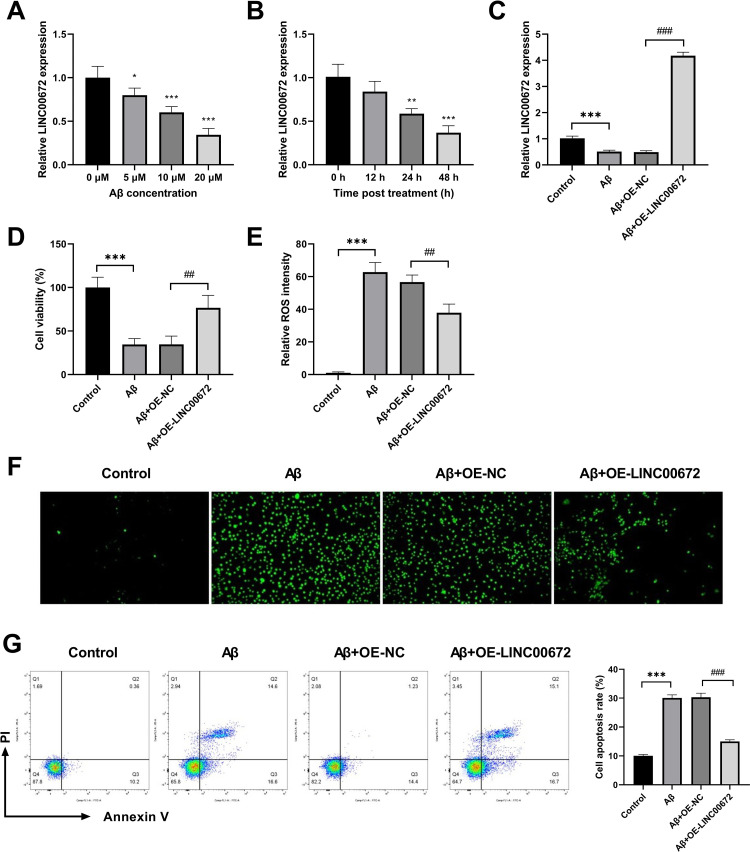
Overexpression of LINC00672 ameliorates Aβ1-42-treated damage in SH-SY5Y cells. (A) SH-SY5Y cells were treated with Aβ1-42 at various concentrations (0, 5, 10, 20 μM) for 24 h, followed by assessing the expression of LINC00672 using quantitative real-time polymerase chain reaction (qRT-PCR). (B) SH-SY5Y cells were treated with 10 μM Aβ1-42 for 0, 12, 24, and 48 h, respectively, followed by assessing the expression of LINC00672 using qRT-PCR. (C) The overexpression efficiency of LINC00672 was detected in SH-SY5Y cells infected with OE-NC or OE-LINC00672 in the presence of Aβ1-42. (D) The viability of SH-SY5Y cells after different treatment was measured using Cell Counting Kit-8 assay. (E) Reactive oxygen species (ROS) levels were examined by corresponding commercial kits. (F) ROS intensity was tested by 2′,7′-dichlorodihydrofluorescein diacetate staining. (G) Flow cytometric analysis was used to detect the apoptosis. ^*^p < 0.05, ^**^p < 0.01, ^***^p < 0.001 *vs.* 0 μM, 0 h or Control; ^##^*p <* 0.01, ^###^*p <* 0.001 *vs.* Aβ+OE-NC.

The SH-SY5Y cells were exposed to Aβ at various concentrations (0, 5, 10, 20 μM) for 24 h, followed by assessing the expression of LINC00672. The expression level of LINC00672 was remarkably decreased in SH-SY5Y cells treated with 10 μM or higher concentrations of Aβ (Fig 1A). Besides, LINC00672 was significantly downregulated in SH-SY5Y cells that received 10 μM Aβ treatment for 24 h or longer (Fig 1B). Therefore, SH-SY5Y cells with 10 μM Aβ treatment for 24 h were used for following experiments.

To substantiate the potential biological function of LINC00672 in AD pathogenesis, LINC00672 was overexpressed in SH-SY5Y cells in the presence of Aβ. We found a significant elevation in Aβ treated SH-SY5Y cells after LINC00672 overexpression (Fig 1C). The viability of SH-SY5Y cells was impaired after Aβ treatment, while was partially recovered by LINC00672 overexpression (Fig 1D). Furthermore, the increased ROS intensity in SH-SY5Y cells induced by Aβ treatment was partially reversed by LINC00672 overexpression (Fig 1E-F). In addition, Aβ treatment exhibited pro-apoptotic effect in SH-SY5Y cells, which was restricted using LINC00672 overexpression (Fig 1G). Collectively, these findings indicated that LINC00672 overexpression alleviated the Aβ-induced damage in SH-SY5Y cells.

### The effects of LINC00672 overexpression in AD mice model

**Fig 2:**
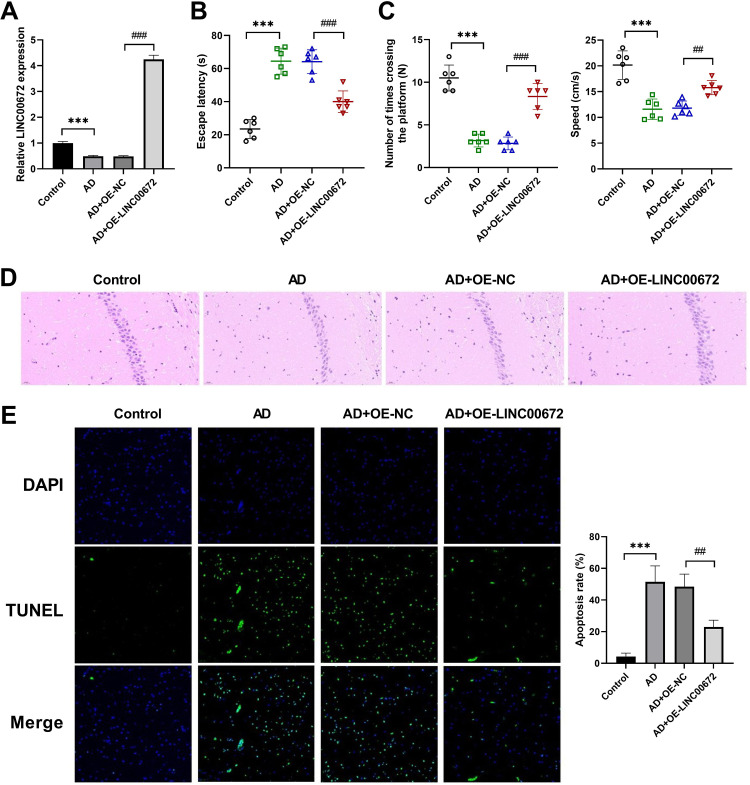
The effects of LINC00672 overexpression in Alzheimer’s disease (AD) mice model. (A) QRT-PCR was used to verify the overexpression efficiency of LINC00672 in vivo. (B-C) The escape latency and the number of platform crossings time spent were determined using Morris water maze test to explore the learning and memory functions of mice after indicated treatment. (D) Effects of streptozotocin treatment on histopathological changes in hippocampus CA1 area were measured by hematoxylin and eosin staining. Amplification: 200 × , scale bar: 50 μm. (E) Cell apoptosis was examined using terminal deoxynucleotidyl transferase dUTP Nick End Labeling assay in the mice hippocampal tissues from control and Model groups. ^***^p < 0.001 *vs.* Control; ^##^*p <* 0.01, ^###^*p <* 0.001 *vs.* AD+OE-NC.

To further elucidating the role of LINC00672 in vivo, LINC00672 overexpression lentivirus were injected into the STZ-treated mice. The expression of LINC00672 was significantly elevated in the AD+OE-LINC00672 group, implying the LINC00672 overexpression mice model was successfully constructed (Fig 2A). The STZ-treated mice displayed prolonged escape latency and a decrease of the number of platform crossings time spent and speed, while these alterations were partially reversed by LINC00672 overexpression (Fig 2B-C). Furthermore, the neuronal cells of the hippocampus CA1 area were loosely arranged with a larger quantity in the control group, while the hippocampal neurons after STZ treatment were arranged in a disorderedly fashion with decreased quantity. In contrast, LINC00672-overexpressed mice exhibited more regular arrangement and increased number of hippocampal neurons (Fig 2D). Additionally, STZ treatment dramatically augmented the apoptosis of neuronal cells in the hippocampus, which were alleviated after LINC00672 overexpression (Fig 2E). The above data illustrated that LINC00672 overexpression could improve spatial memory and attenuate neuronal death in the STZ-treated AD mice.

### LINC00672 overexpression promotes autophagy in Aβ-treated cells

**Fig 3:**
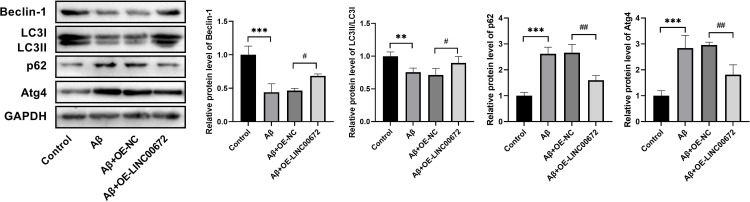
LINC00672 overexpression affects autophagy in Aβ1-42-treated cells. Western blot was performed to detect the protein expressions of Beclin-1, LC3II/LC3I levels, and P62 in SH-SY5Y cells after different treatment. ^**^p < 0.01, ^***^p < 0.001 *vs.* Control; ^#^*p <* 0.05, ^##^*p <* 0.01 *vs.* Aβ+OE-NC.

In order to unravel whether LINC00672 overexpression affected autophagy in AD progression, we examined the hallmarks related to autophagy. The Beclin-1 and LC3II/LC3I levels were declined while p62 and Atg4 levels were elevated in Aβ group, which were partially abolished using LINC00672 overexpression (Fig 3). Taken together, overexpression of LINC00672 contributed to reducing the suppressed activation of autophagy in Aβ-treated SH-SY5Y cells.

### LINC00672 enhances autophagy by targeting GPNMB in Aβ-treated cells

To further check whether LINC00672 modulated autophagy via targeting GPNMB in AD cell model. Western blot demonstrated that GPNMB was significantly downregulated in the Aβ group compared to the Control group, with no statistically significant difference observed between the Aβ group and Aβ+OE-NC group in terms of GPNMB expression; the expression level of GPNMB was elevated in Aβ+OE-LINC00672 compared to Aβ+OE-NC group (Fig 4A). Furthermore, GPNMB silencing led to a reduction in GPNMB, Beclin-1 and LC3II/ LC3I, and an increase of p62 and Atg4 levels in LINC006720-overexpressed AD cell model (Fig 4B), implying LINC00672 could enhance autophagy by targeting GPNMB in Aβ-stimulated SH-SY5Y cells.

**Fi 4:**
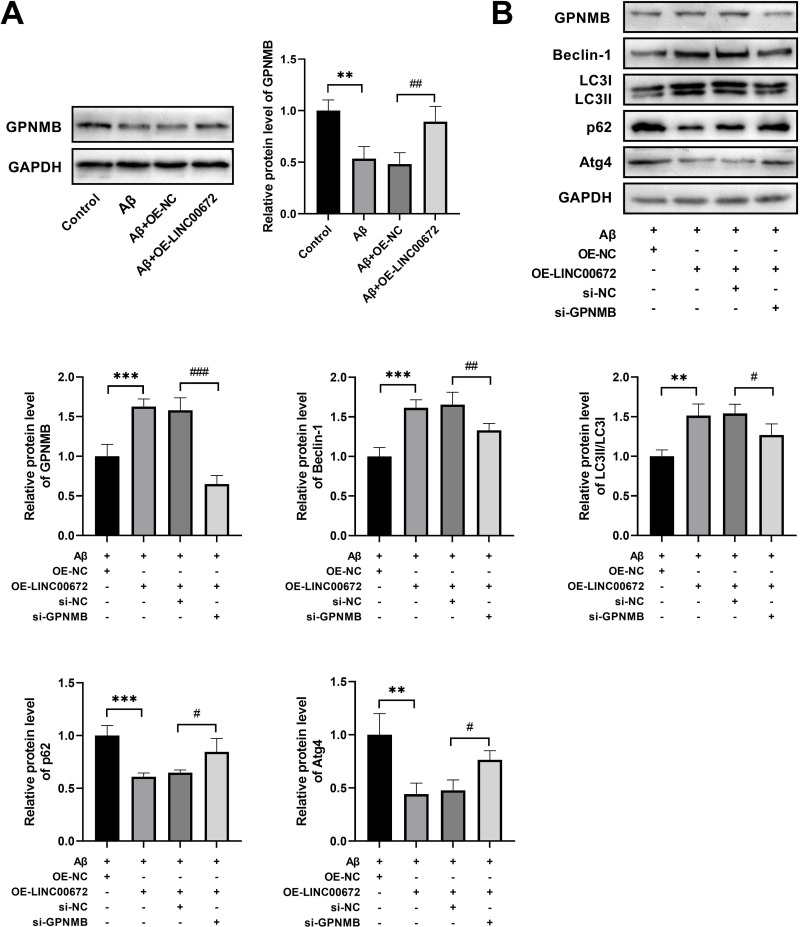
LINC00672 enhances autophagy by targeting GPNMB in AD cell model. (A) The relative protein levels of GPNMB in AD cell model. ^**^p < 0.01, *vs.* Control; ^##^*p <* 0.01, Aβ+OE-NC. (B) The protein expressions of GPNMB, Beclin-1, LC3II/ LC3I levels, and P62 in Aβ-stimulated SH-SY5Y cells combined with LINC00672 overexpression were determined by western blot analysis. ^**^p < 0.01, ^***^p < 0.001 *vs.* Aβ+OE-NC; ^#^*p <* 0.05, ^##^*p <* 0.01, ^###^*p <* 0.001 *vs.* Aβ+OE-LINC00672+si-NC.

### LINC00672 overexpression alleviates Aβ-treated neuronal damage through regulating GPNMB

**Fig 5:**
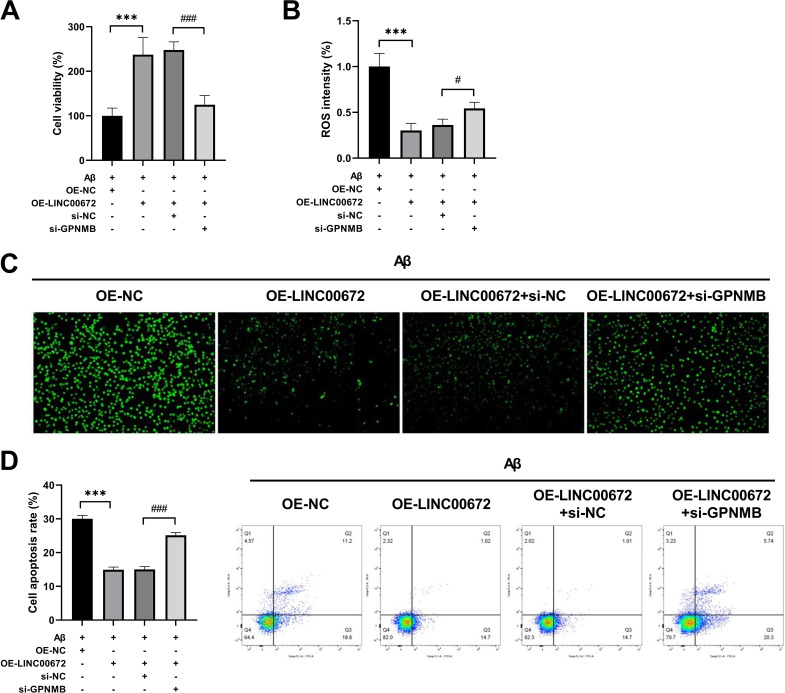
LINC00672 overexpression alleviates A β-treated neuronal damage through regulating GPNMB. (A) The Cell Counting Kit-8 assay was used to measure the viability of SH-SY5Y cells in Aβ+OE-NC, Aβ+OE-LINC00672, Aβ+OE-LINC00672+si-NC, and Aβ+OE-LINC00672+si-GPNMB groups. (B) ROS levels in SH-SY5Y cells of each group were evaluated using corresponding commercial kits. (C) ROS intensity in SH-SY5Y cells of each group was tested by 2′,7′-dichlorodihydrofluorescein diacetate staining. (D) The apoptotic rate was measured by flow cytometric analysis. ^***^p < 0.001 *vs.* Aβ+OE-NC; ^#^*p <* 0.05, ^###^*p <* 0.001 *vs.* Aβ+OE-LINC00672+si-NC.

We next validated the effects of GPNMB silencing in Aβ-treated neuronal damage. We found that GPNMB knockdown diminished the improved effect of LINC00672 overexpression on viability in Aβ-stimulated SH-SY5Y cells (Fig 5A). Besides, the reduced ROS intensity caused by LINC00672 overexpression in Aβ-treated SH-SY5Y cells was increased after GPNMB silencing (Fig 5B-C). In addition, the apoptotic rate was notably higher in Aβ+OE-LINC00672+si-GPNMB than that in Aβ+OE-LINC00672+si-NC group (Fig 5D). The findings further demonstrated that LINC00672 overexpression could alleviate Aβ-stimulated neuronal injury through regulating GPNMB.

## Discussion

AD is complex and multifactorial in etiopathogenesis with substantially increasing incidence and prevalence that represents a growing encumbrance to health concern in aging populations [24,25]. Hence, further exploration of AD epidemiology could facilitate the discovery of novel therapeutic modalities to target both the onset and progression of AD. In the present study, overexpression of LINC00672 could ameliorate Aβ-stimulated damage in SH-SY5Y cells, and improve spatial memory and hippocampal neuronal death in the STZ-treated AD mice. Furthermore, overexpressed LINC00672 reduced the suppressed activation of autophagy in AD cell model. Mechanistically, LINC00672 upregulation alleviated Aβ-stimulated neuronal injury and enhanced autophagy by targeting GPNMB.

LncRNAs have emerged as preeminent regulatory molecules involved in various neurodegenerative illnesses including AD [26]. LINC01311 upregulation displays the protective effects towards Aβ-induced apoptosis and proliferation slowdown in SH-SY5Y cells [27]. Overexpression of lncRNA WT1-AS facilitates the proliferation and restricts the apoptosis of Aβ-treated neuroblastoma cells [28]. *Gu et al.* have found that lncRNA Rpph1 overexpression is able to alleviate the neuronal damage in SK-N-SH cells [29]. LINC00672 is recently found to be negatively correlated with the Braak stage linked to AD [16]. Nonetheless, the accurate biological capabilities of LINC00672 in AD remain unclear. In the present study, we found that the expression of LINC00672 was dose-dependently decreased in SH-SY5Y cells following Aβ treatment. Moreover, LINC00672 overexpression could mitigate the Aβ-induced damage in SH-SY5Y cells, as evidenced by recovered viability, decreased ROS intensity, and reduced apoptosis. Besides, LINC00672 also improved spatial memory and repressed hippocampal neuronal death in the STZ-treated AD mice. These data implied that LINC00672 conferred the protective effects against AD.

Accumulating evidence has highlighted targeting autophagy holds great potential in modulation of neurodegenerative disorders [30]. Autophagy dysregulation is found to have associations with medium spiny neurons degeneration in Huntington disease, and enhancing autophagy is deemed as the potential approach for protecting neurons [31]. BDNF exhibits neuroprotective effects in PD mice, which is imparted by promoting autophagy [32]. Overexpression of miR-331-3p and miR-9-5p can diminish autophagic activity in SH-SY5Y cell line [33]. On the contrary, it is reported that autophagy is impaired in patients with AD, which is possibly restored by PACAP [34]. The autophagy levels were escalated with high-concentration of Aβ25–35 treatment in AD cell model, while the elevated expression of FoxG1 possesses the capacity to activate the autophagy pathway [35]. Consistently, in this research, our data demonstrated that the Beclin-1 and LC3II/LC3I levels were declined while p62 level was elevated in Aβ-treated SH-SY5Y cells, while were partially reversed by LINC00672 overexpression These findings illustrated that overexpression of LINC00672 contributed to the activation of autophagy in AD cell model.

GPNMB has prominent implications in neurological researches, which has gained extensive attention. LncRNA MALAT1 modulates the cell proliferation and apoptosis in PD cell model, which is possibly mediated by the miR-135b-5p/GPNMB axis [36]. GPNMB knockdown is capable to facilitate neuronal loss, apoptosis, and neuroinflammation of the hippocampus in pilocarpine-induced epilepsy [17]. Furtherly, GPNMB exerts its function possibly linked to the involvement in autophagy. It is reported that GPNMB can enhance autophagy following spinal cord injury, thereby alleviating the inflammatory response and hindering cell apoptosis [37]. Furthermore, high level of GPNMB is found in the brain of AD mice, which may help the clearance of Aβ and improve AD-like behaviors by promoting autophagy [21]. Herein, we revealed that GPNMB silencing partially counteracted the effects of LINC00672 overexpression on viability, ROS levels, and apoptosis. Furthermore, GPNMB knockdown restrained the activation of autophagy in Aβ-stimulated SH-SY5Y cells after LINC00672 overexpression, implying GPNMB silencing possibly as a negative regulator of autophagy towards LINC00672 overexpression during AD.

To summarize, overexpression of LINC00672 exerted the protective role against AD. Furthermore, LINC00672 upregulation represented a contributive factor to the activation of autophagy in AD cell model. In addition, LINC00672 overexpression repressed Aβ-stimulated neuronal injury and accelerated autophagy, while this situation was weakened by the GPNMB knockdown. Taken together, LINC00672 upregulates GPNMB to promote autophagy, exerting a protective effect in the AD progression. Findings of this study may deepen the understanding of AD etiopathogenesis, and also offer meaningful references for further exploration of AD development.

## Suppovrting information

S1 Table The sequences of primers and siRNAs about target genes for quantitative real-time polymerase chain reaction. (DOCX)

S1 File This file contains the original raw data in Excel format, including all experimental measurements and observations collected during the study. Data are organized by experimental groups and correspond to the analyses presented in the main figures and results section. (XLSX)

S2 File Original WB blots. This file provides the complete, uncropped Western blot (WB) images corresponding to all WB results described in the manuscript. Original blot images are presented without modifications to ensure transparency and reproducibility. (PDF)
