# Supplementary material for: Overexpression of LINC00672 promotes autophagy in Alzheimer’s disease by upregulating GPNMB
Source: PLoS One. 2025 May 14;20(5):e0322708. doi: 10.1371/journal.pone.0322708 (PMC12077738; doi:10.1371/journal.pone.0322708)
Supplement: S1 Table — (DOCX) [file pone.0322708.s001.docx]

**Table S1**

The sequences of primers and siRNAs about target genes for quantitative real-time polymerase chain reaction (qRT-PCR).

| Name | Sequences (5’-3’) |
| --- | --- |
| GAPDH-F | CTCATGACCACAGTCCATGC |
| GAPDH-R | TTCAGCTCTGGGATGACCTT |
| LINC00672-F | GCGAAGAAGGCAGTCAGGAGGA |
| LINC00672-R | ACCAACCACAGCCAACCAATCAC |
| si-GPNMB-F | GGAGCUGAGUAGGAUUCCUGAUGAA |
| si-GPNMB-R | UUCAUCAGGAAUCCUACUCAGCUCC |
| si-NC-F | UUCUCCGAACGUGUCACGUTT |
| si-NC-R | ACGUGACACGUUCGGAGAATT |
