# Supplementary material for: Overexpression of LINC00672 promotes autophagy in Alzheimer’s disease by upregulating GPNMB
Source: PLoS One. 2025 May 14;20(5):e0322708. doi: 10.1371/journal.pone.0322708 (PMC12077738; doi:10.1371/journal.pone.0322708)

Fig3-Beclin-1

(include multiple exposures)

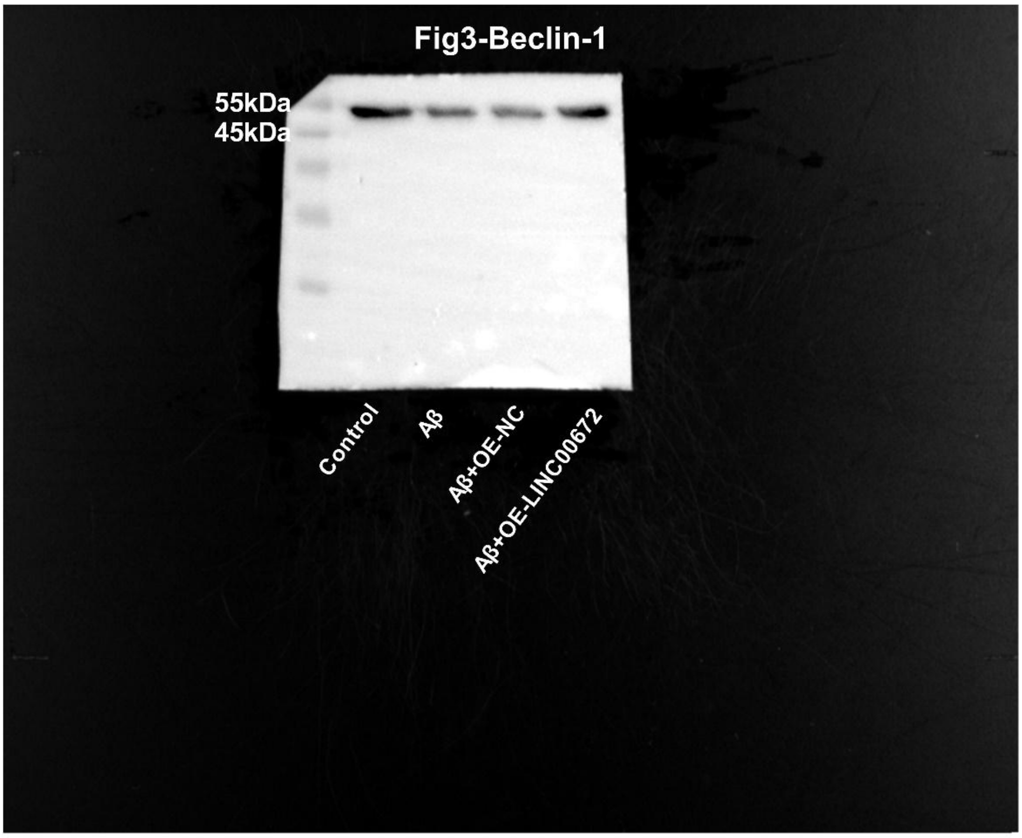

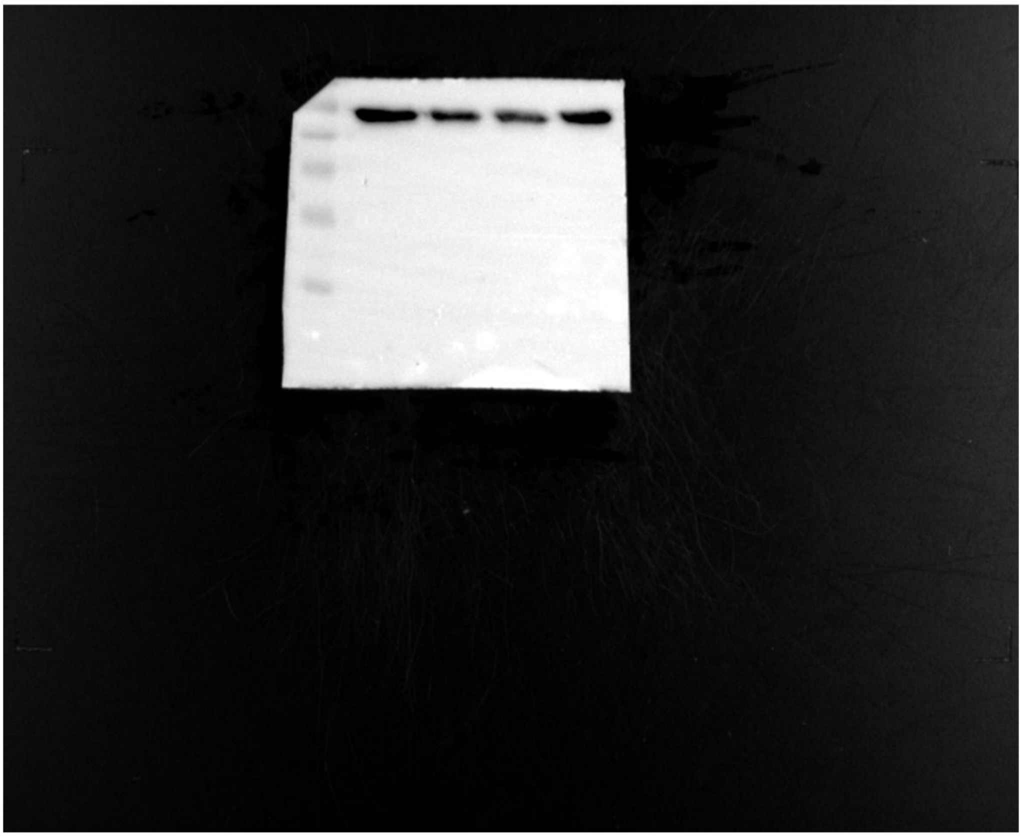

Fig3-LC3I、LC3II

(include multiple exposures)

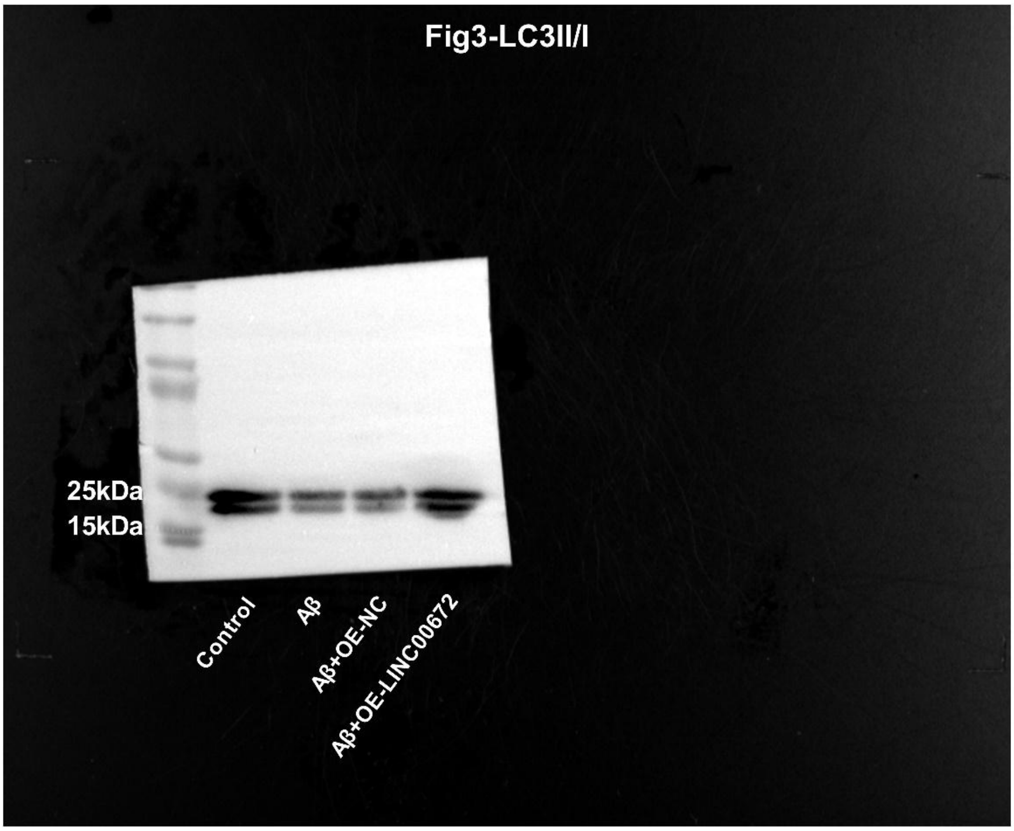

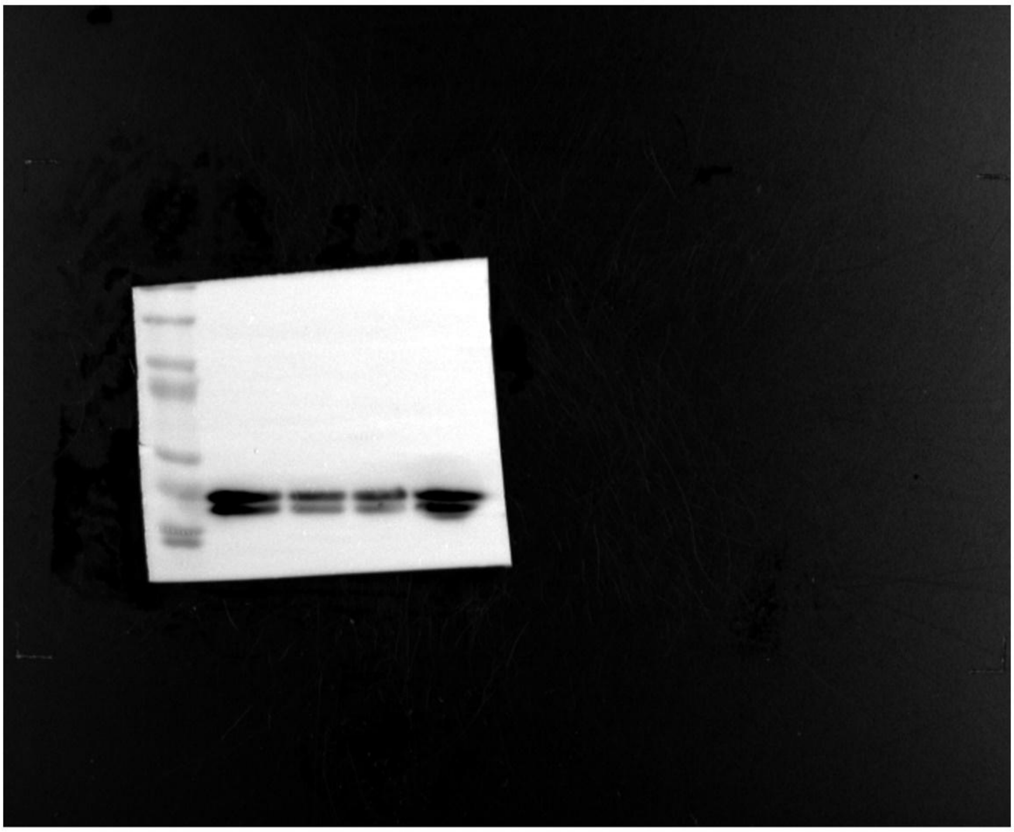

Fig3-p62

(include multiple exposures)

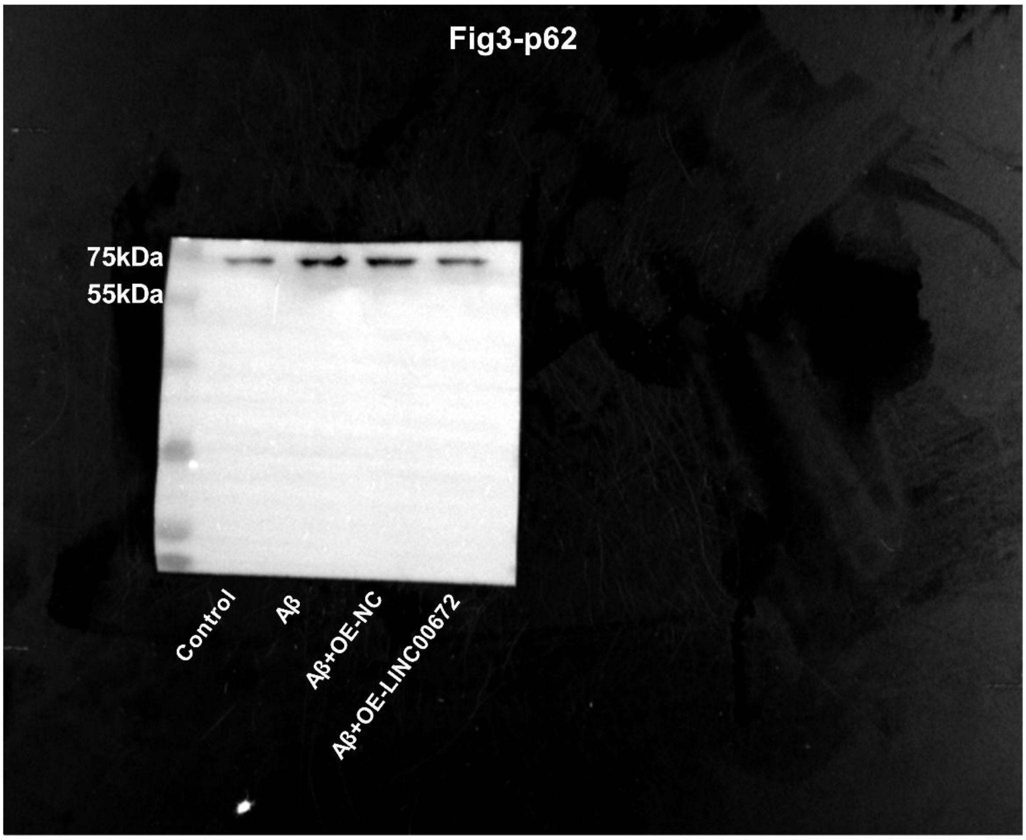

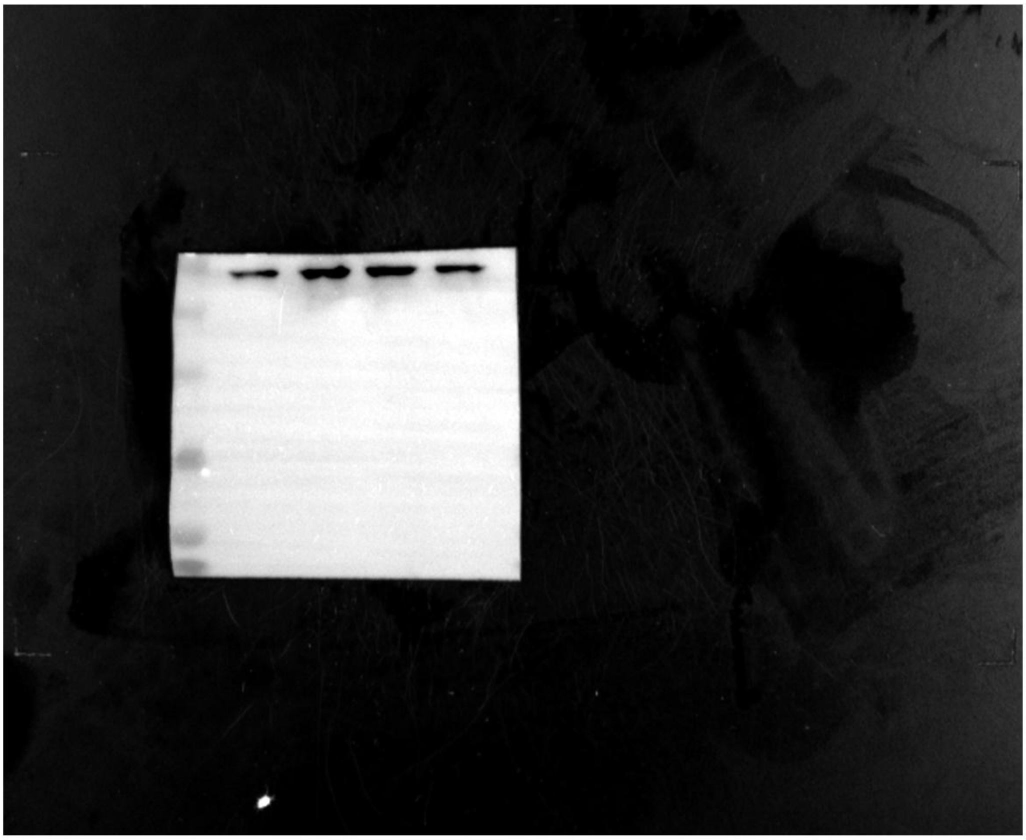

Fig3-Atg4

(include multiple exposures)

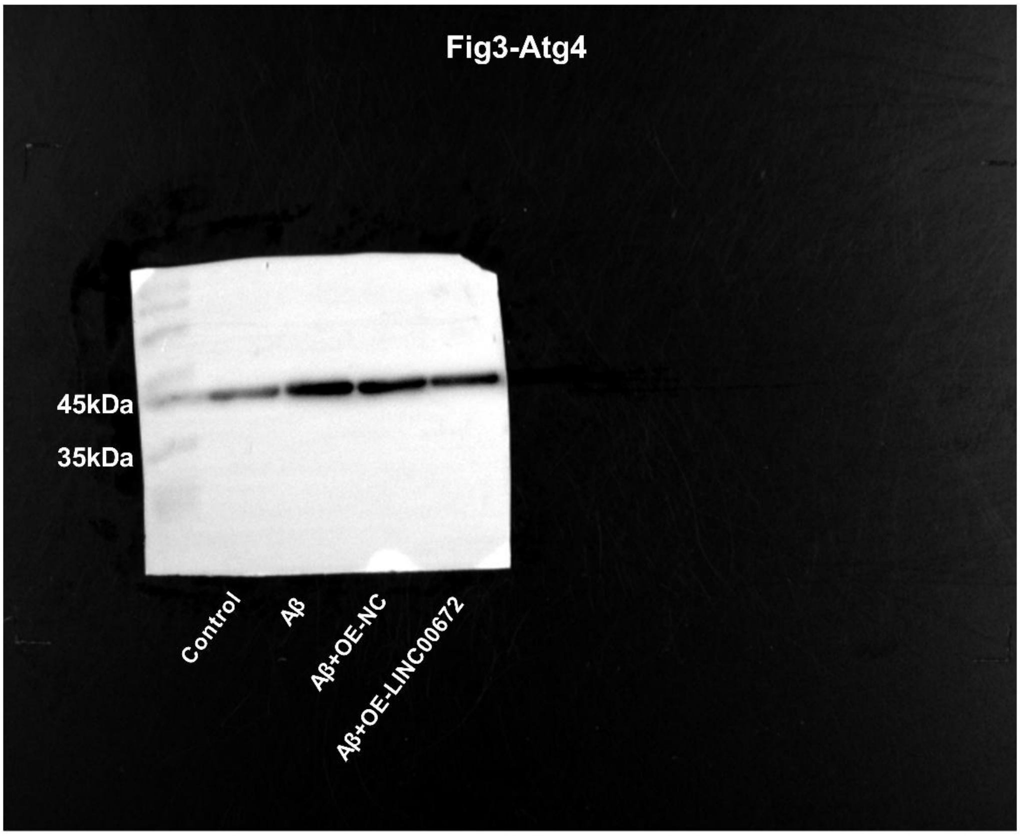

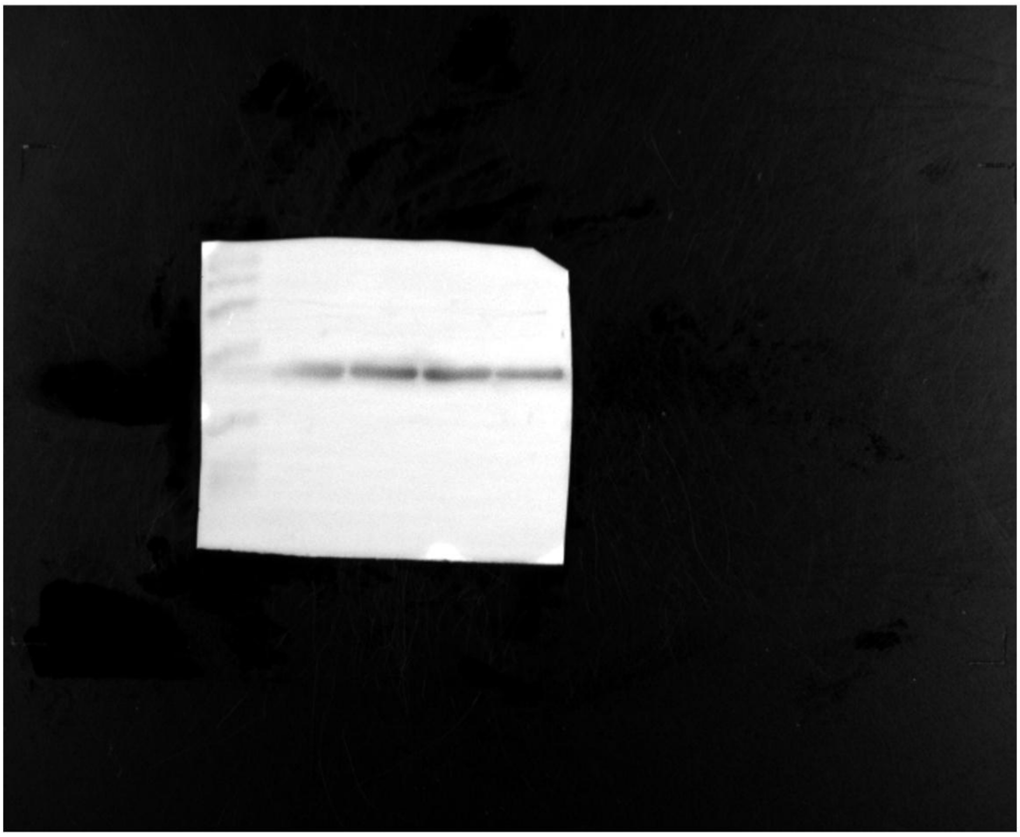

Fig3-GAPDH

(include multiple exposures)

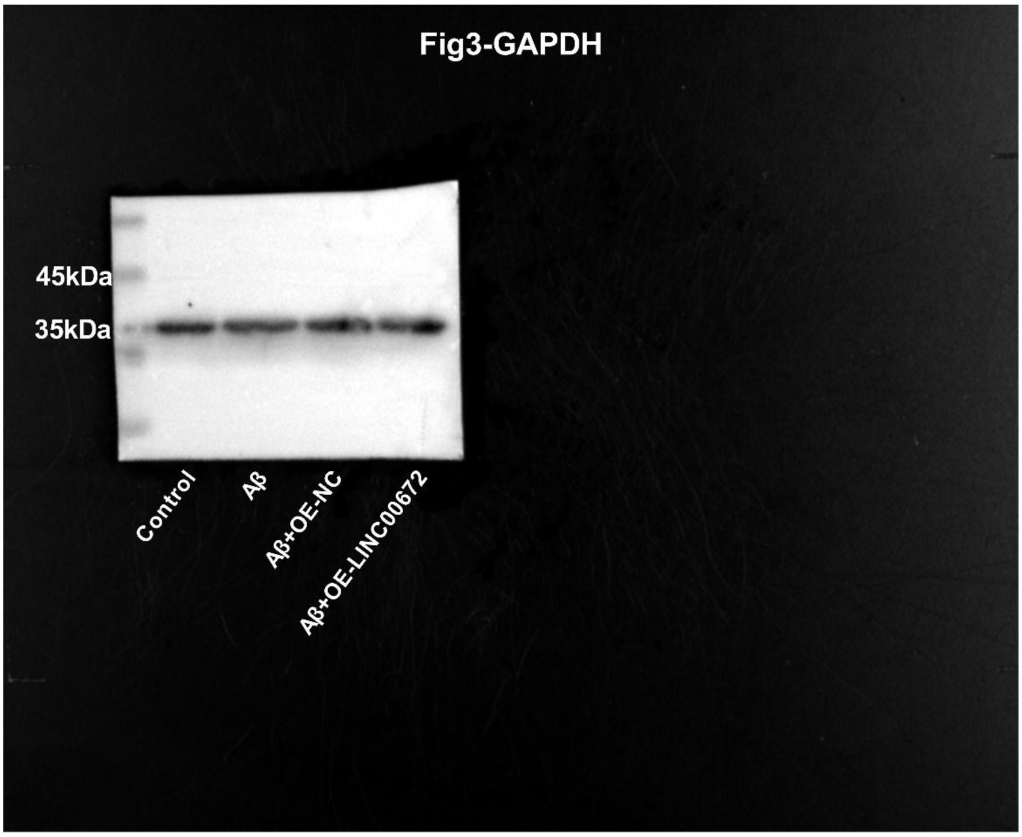

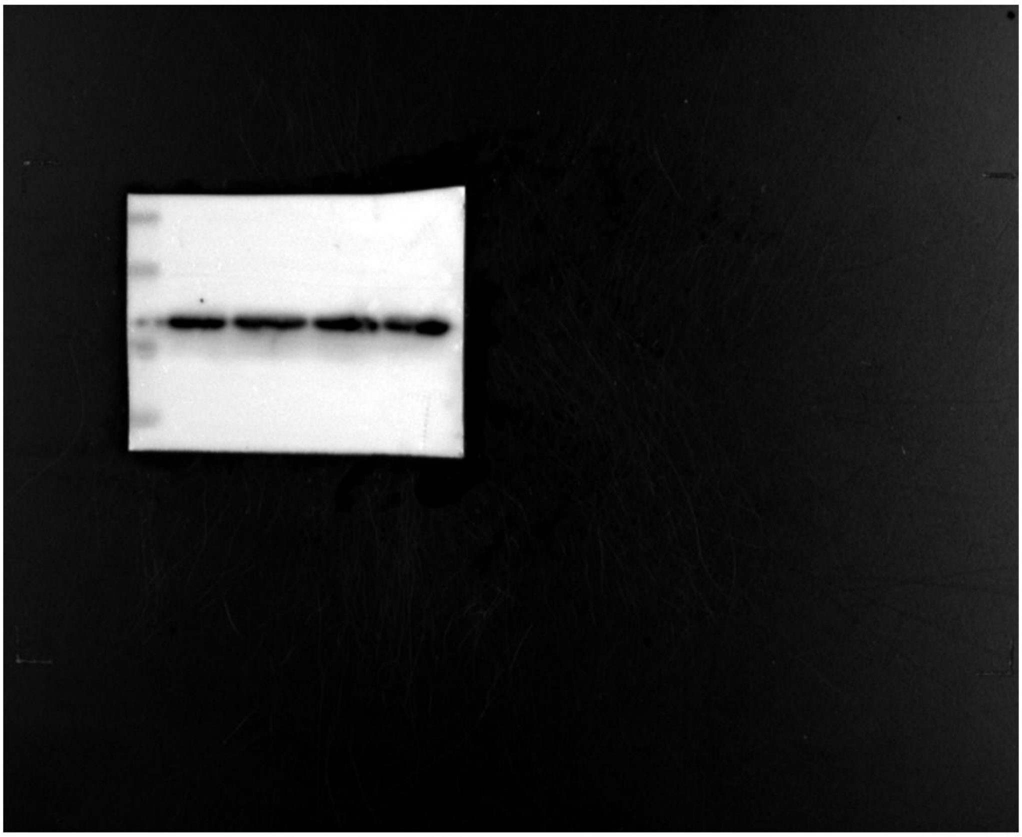

Fig4A-GPNMB

(include multiple exposures)

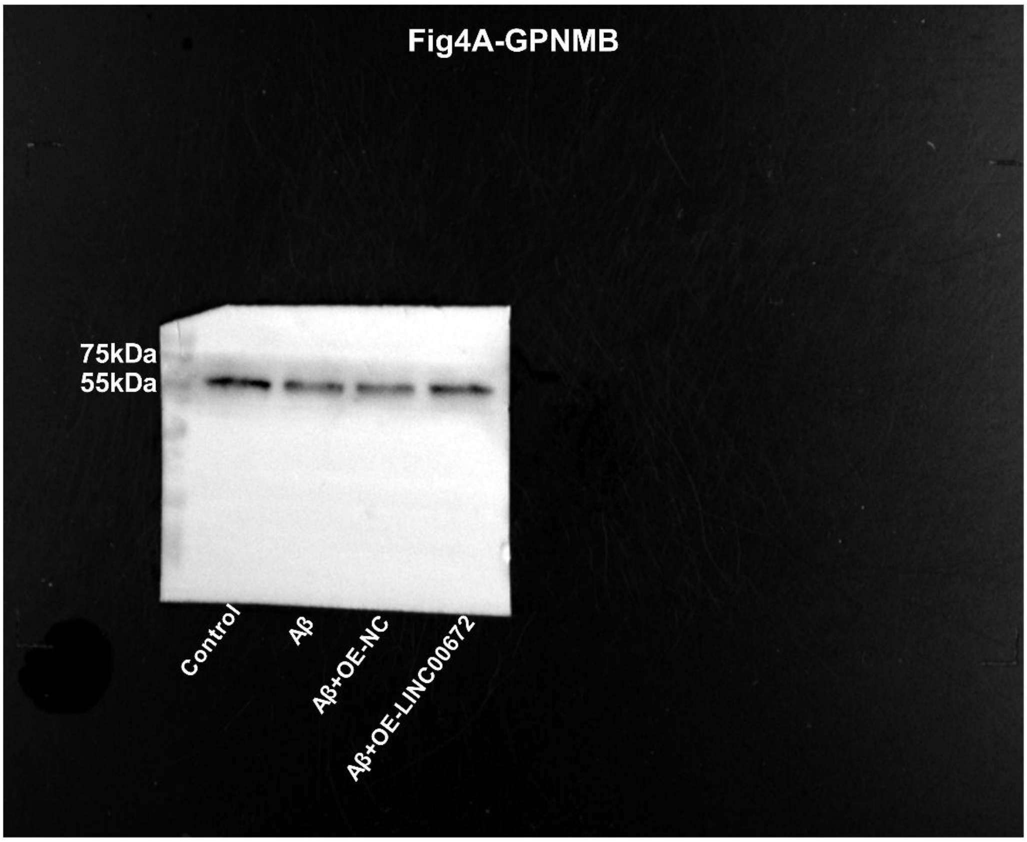

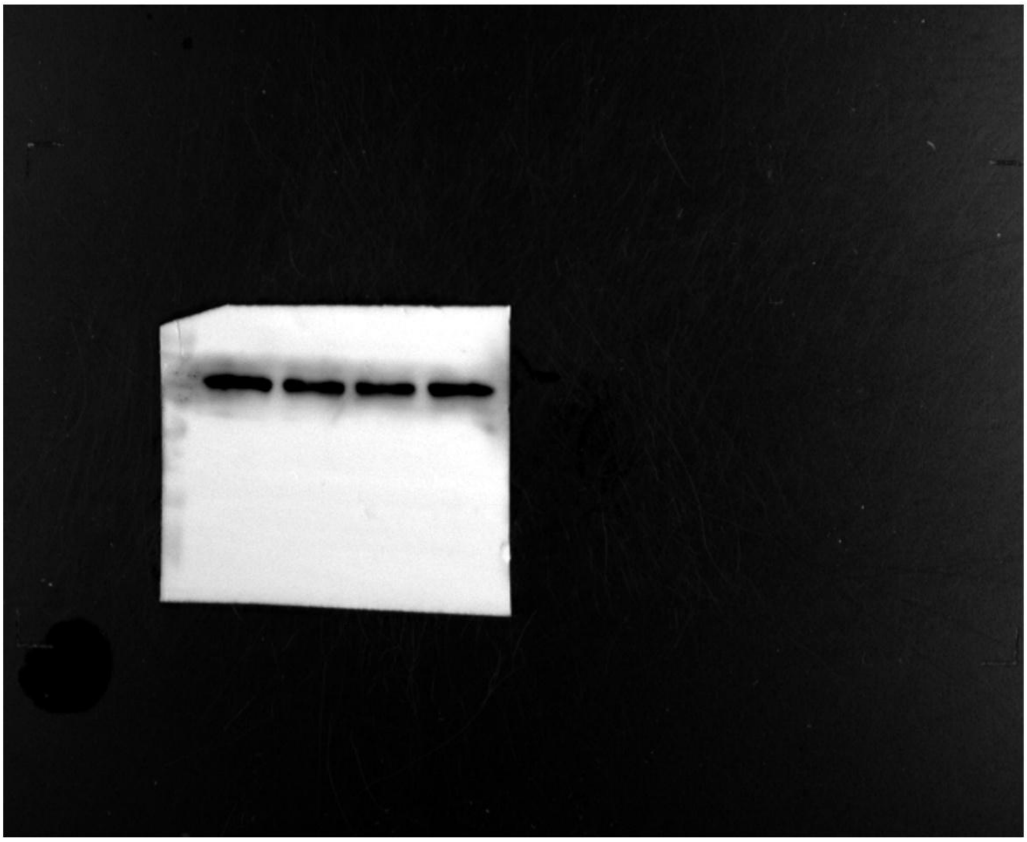

Fig4A-GAPDH

(include multiple exposures)

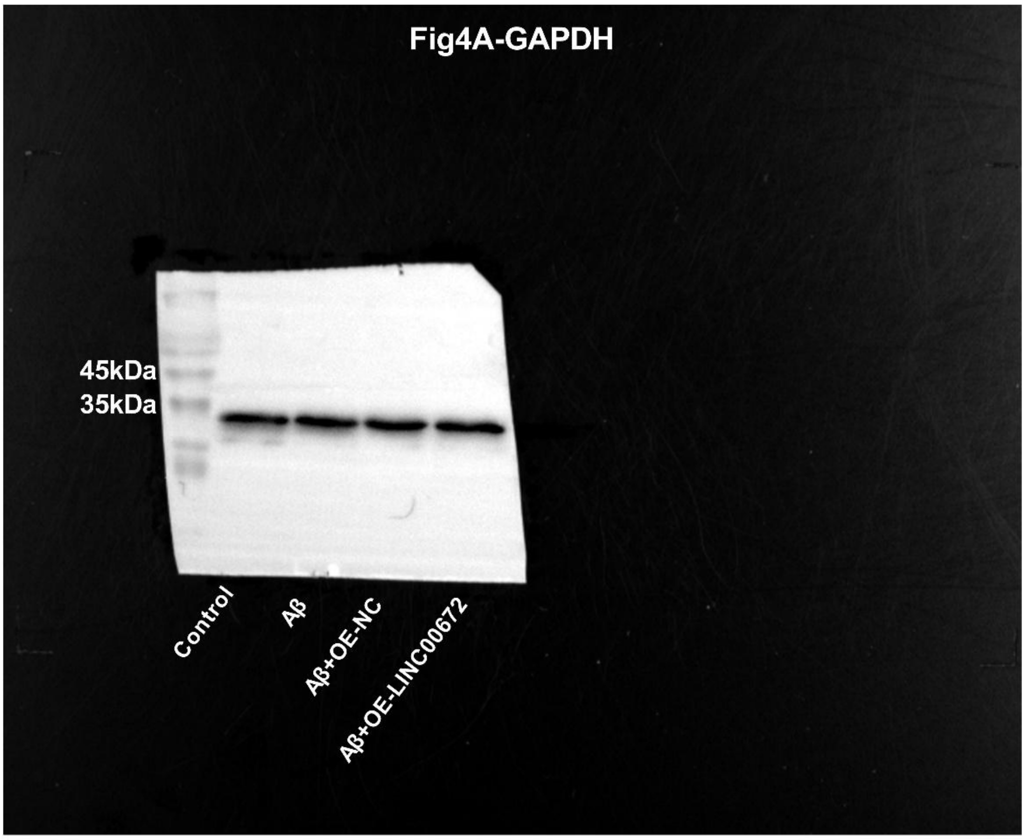

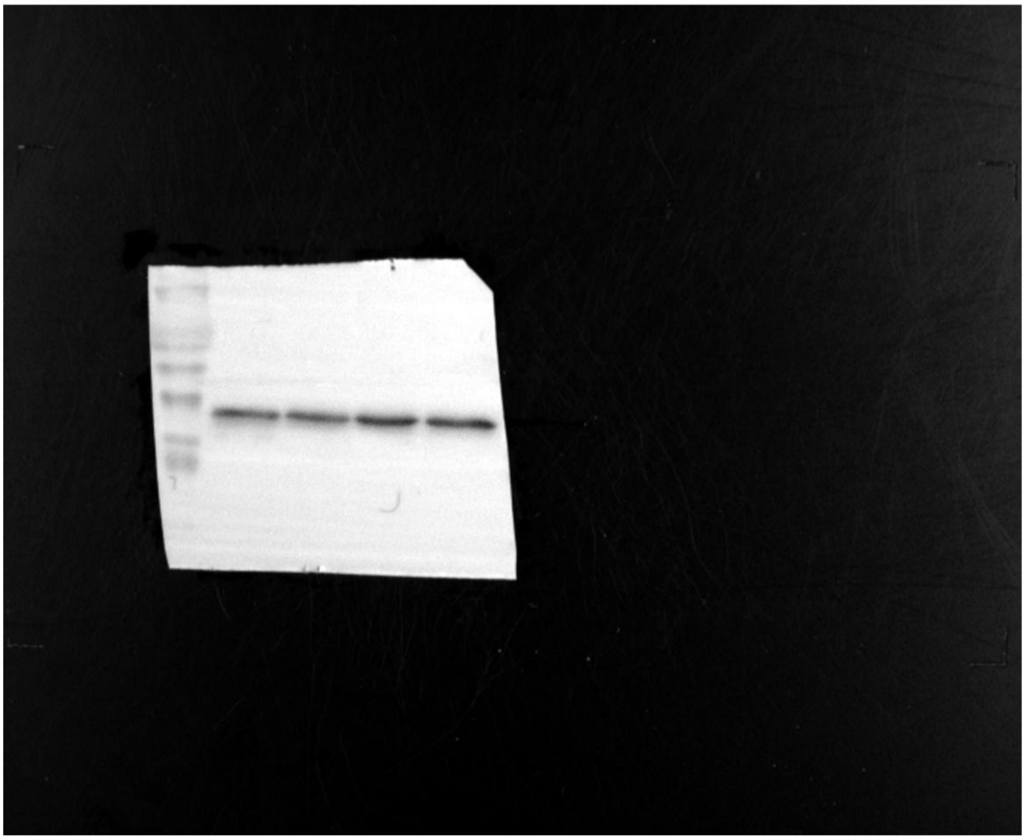

Fig4B-GPNMB

(include multiple exposures)

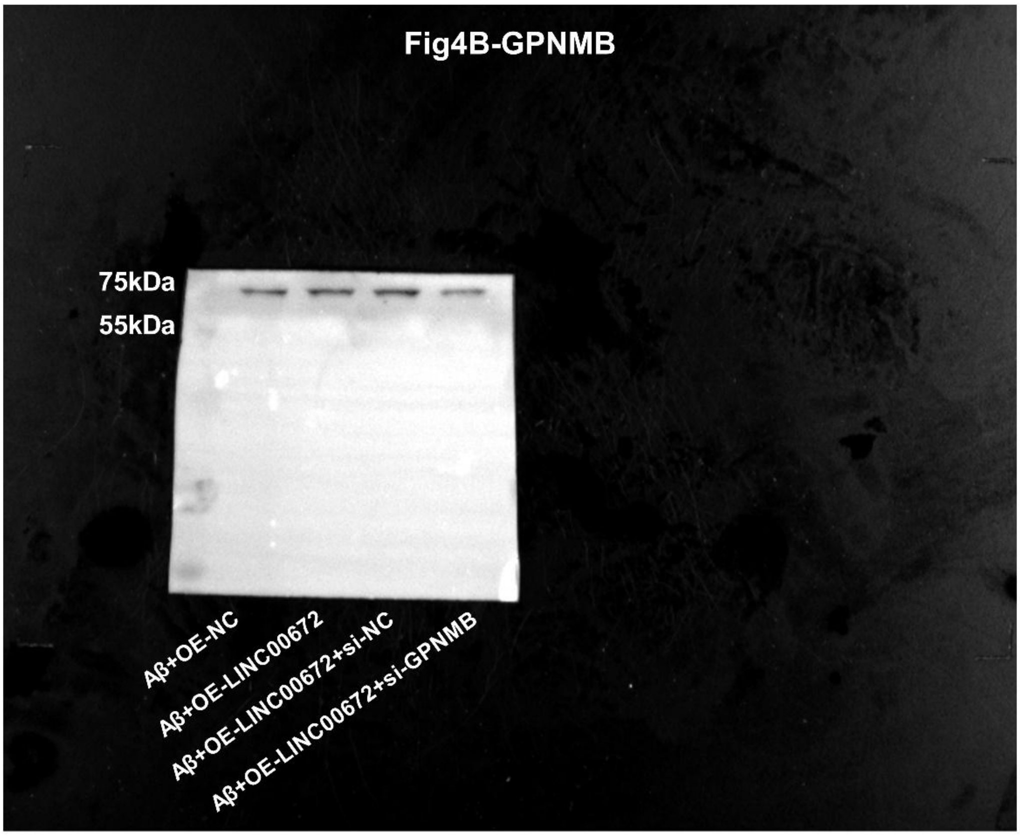

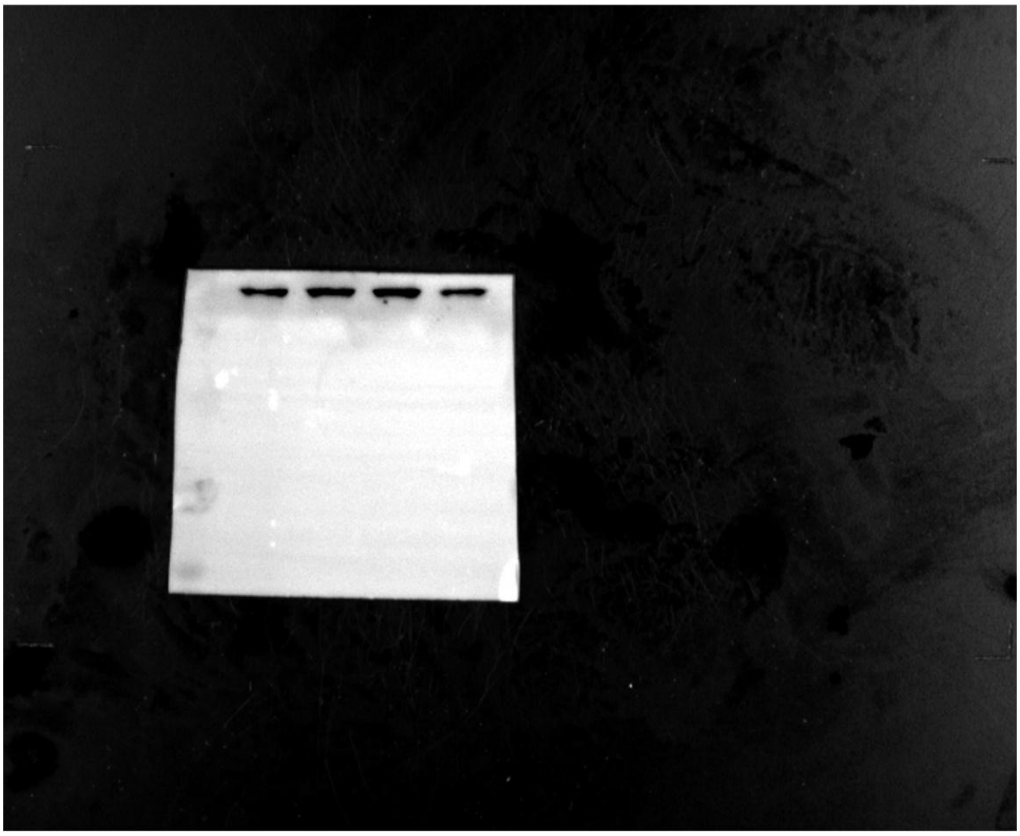

Fig4B-Beclin-1

(include multiple exposures)

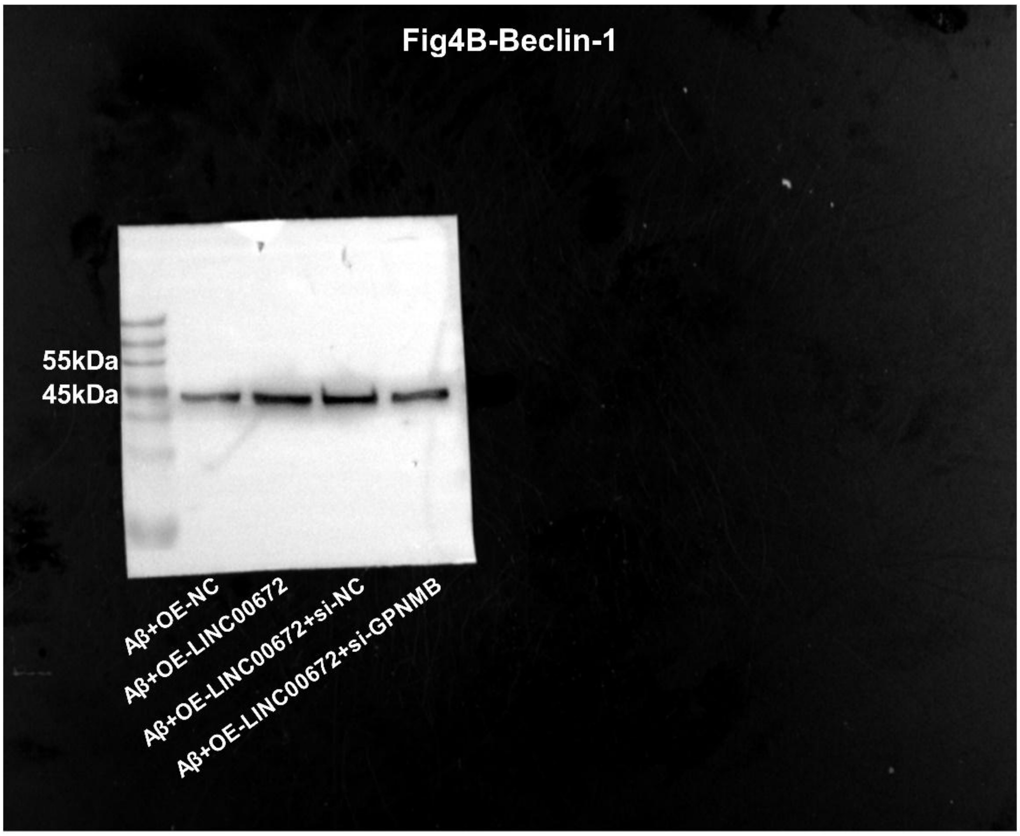

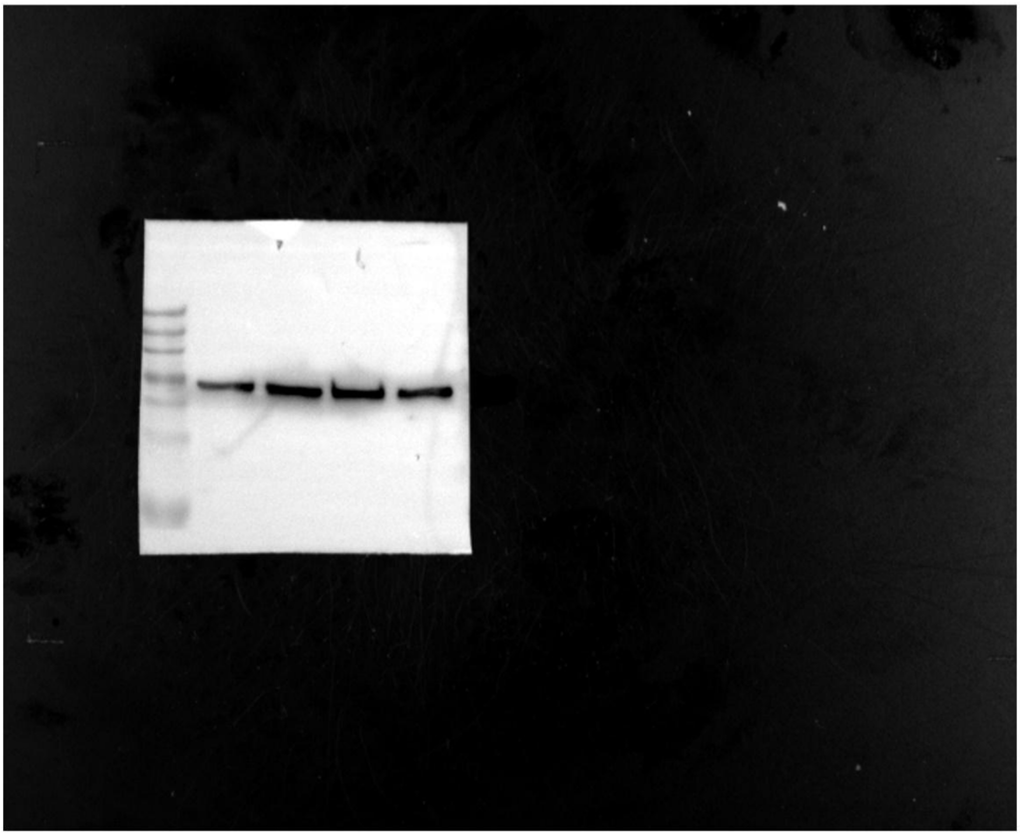

Fig4B-LC3I、LC3II

(include multiple exposures)

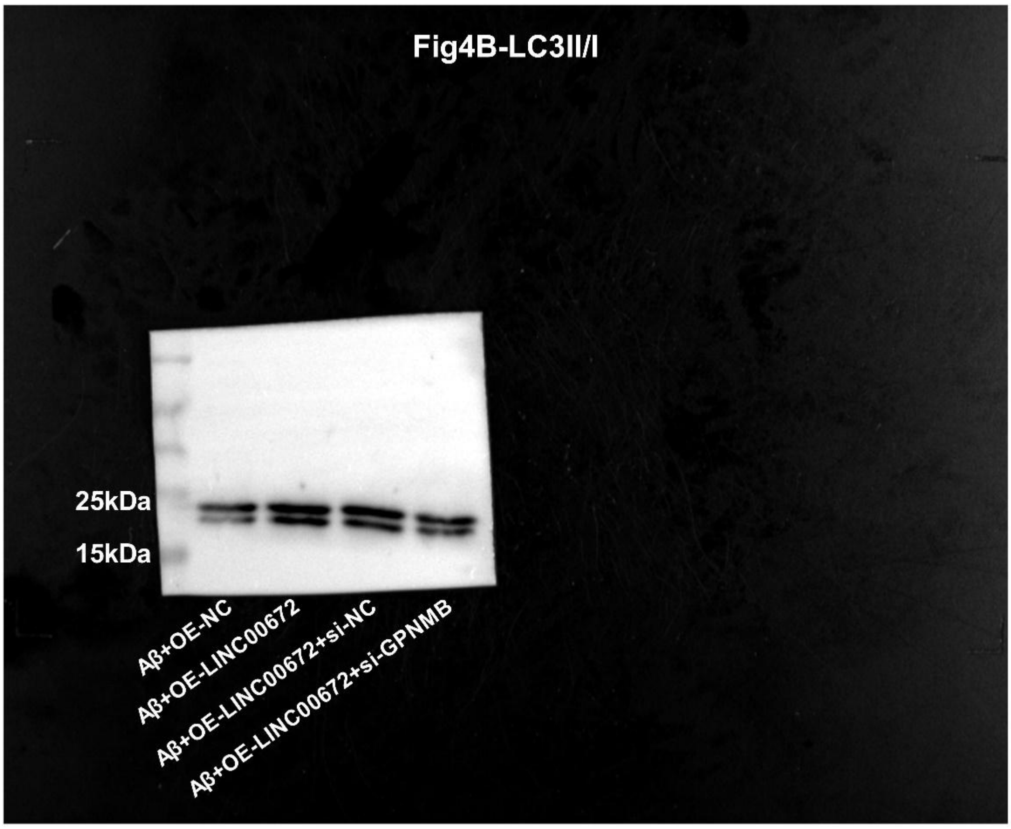

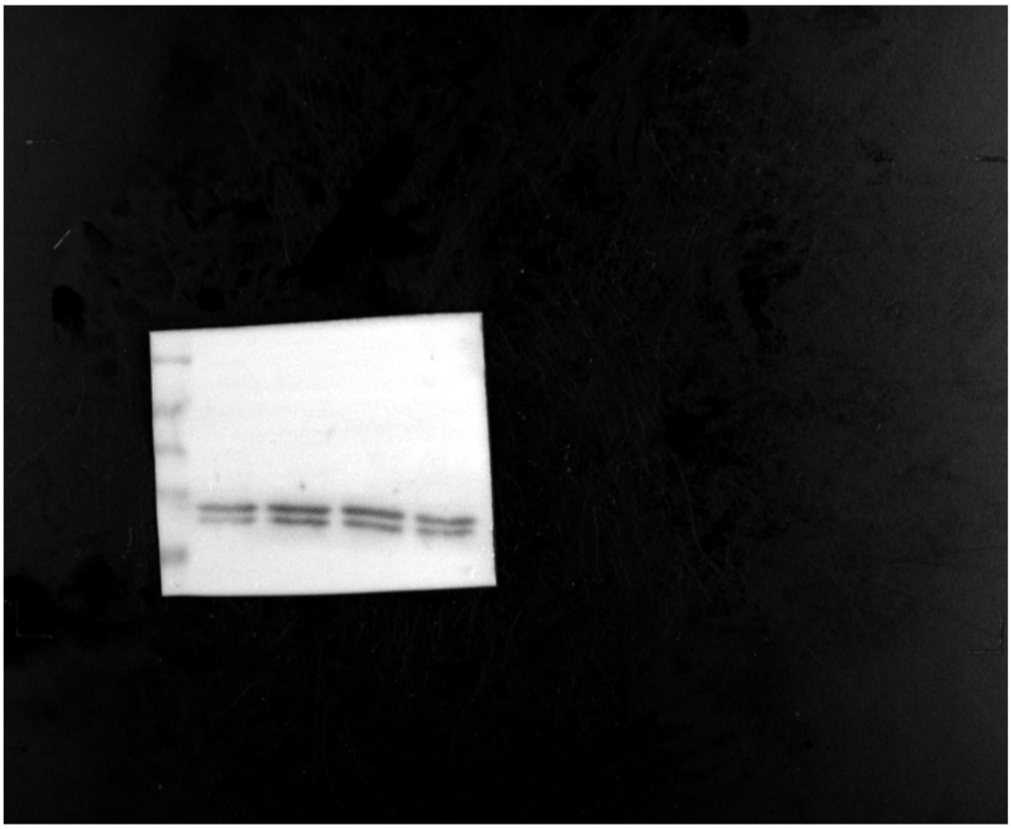

Fig4B-p62

(include multiple exposures)

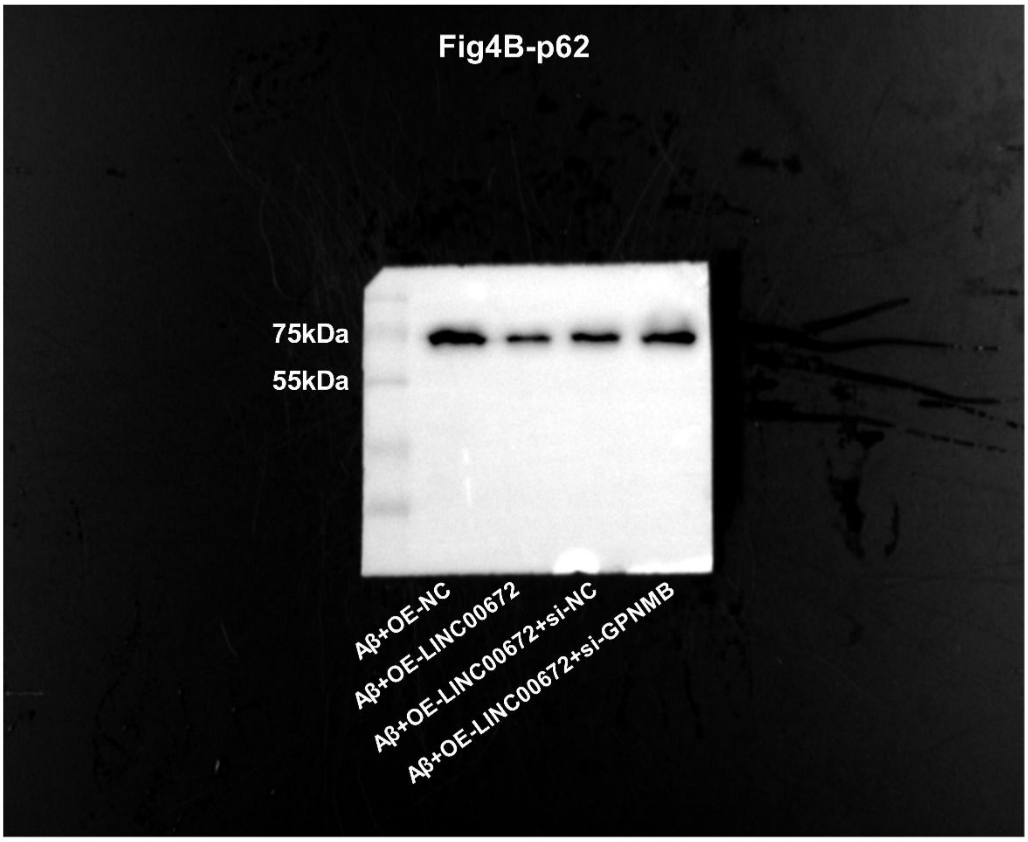

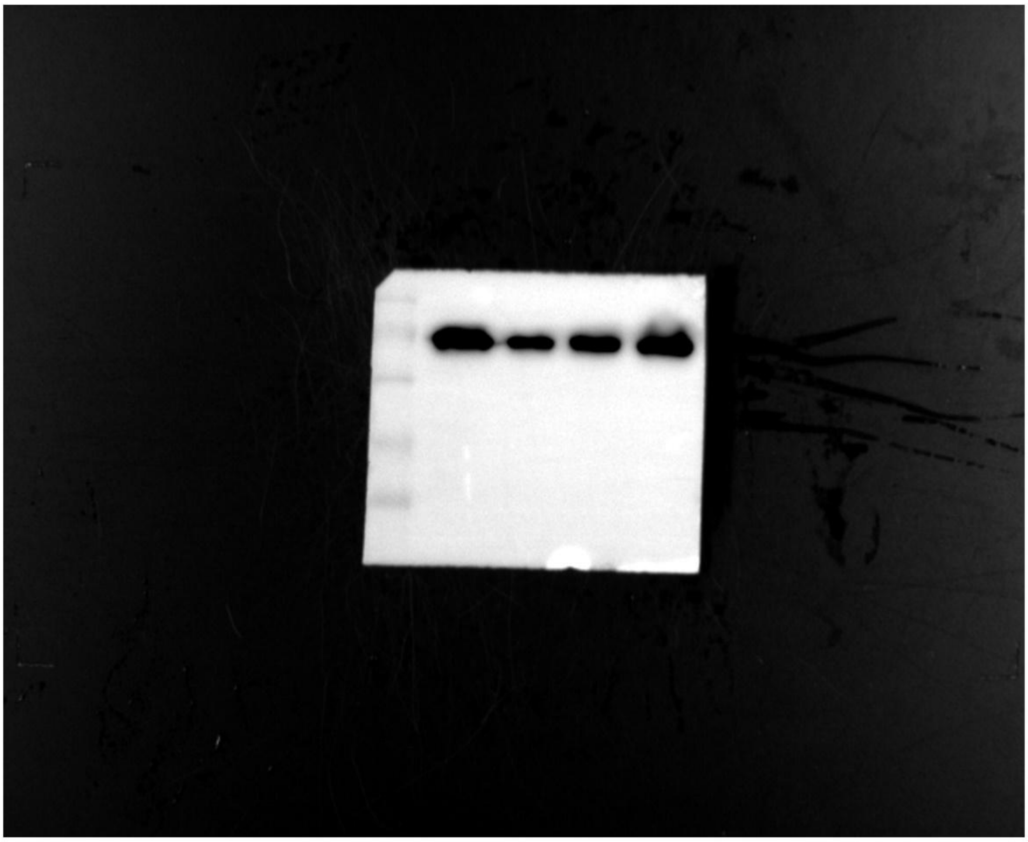

Fig4B-Atg4

(include multiple exposures)

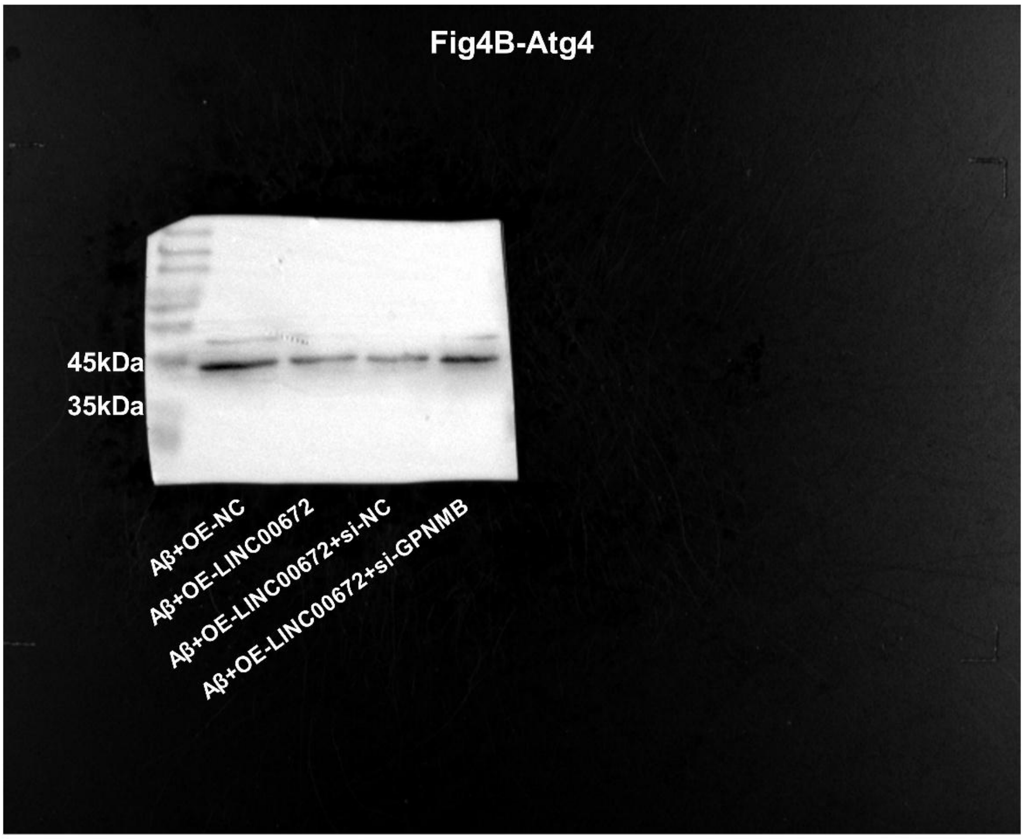

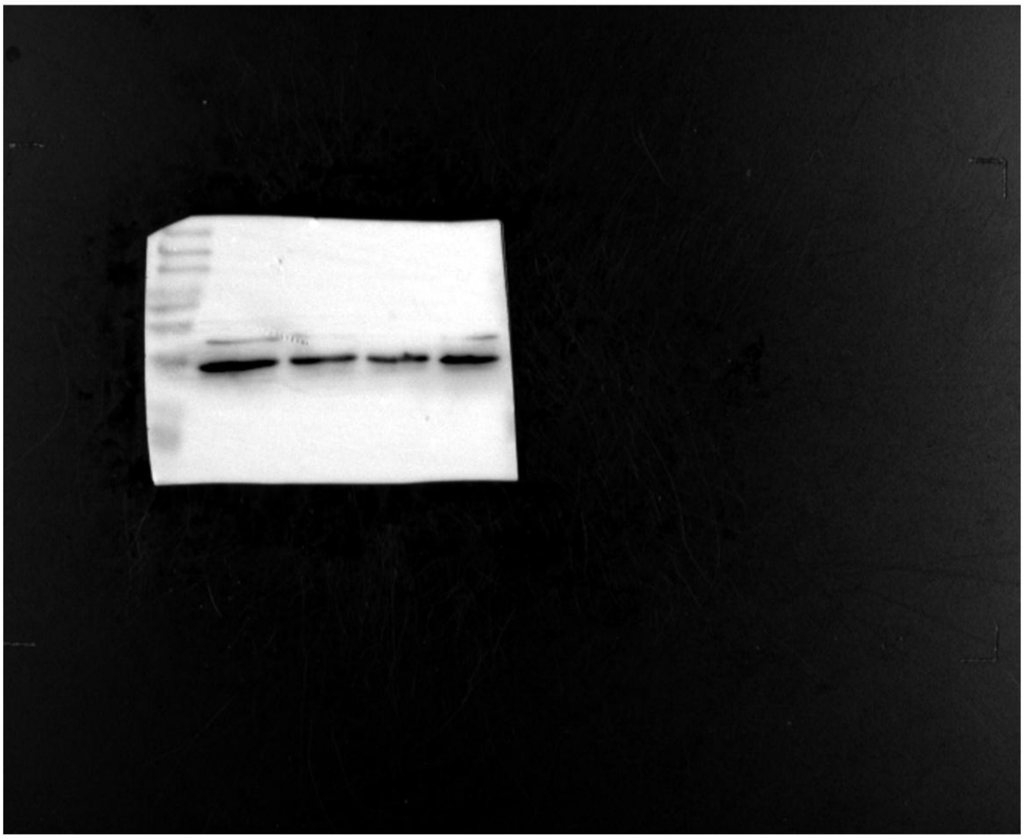

Fig4B-GAPDH

(include multiple exposures)

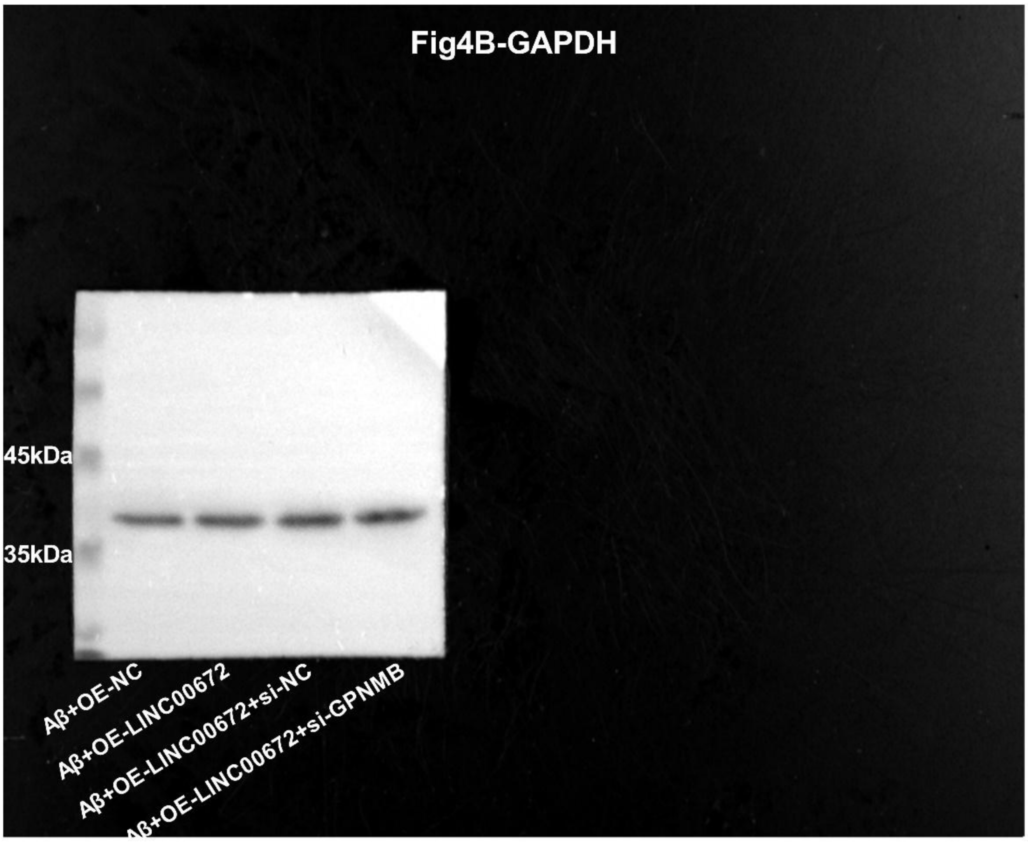

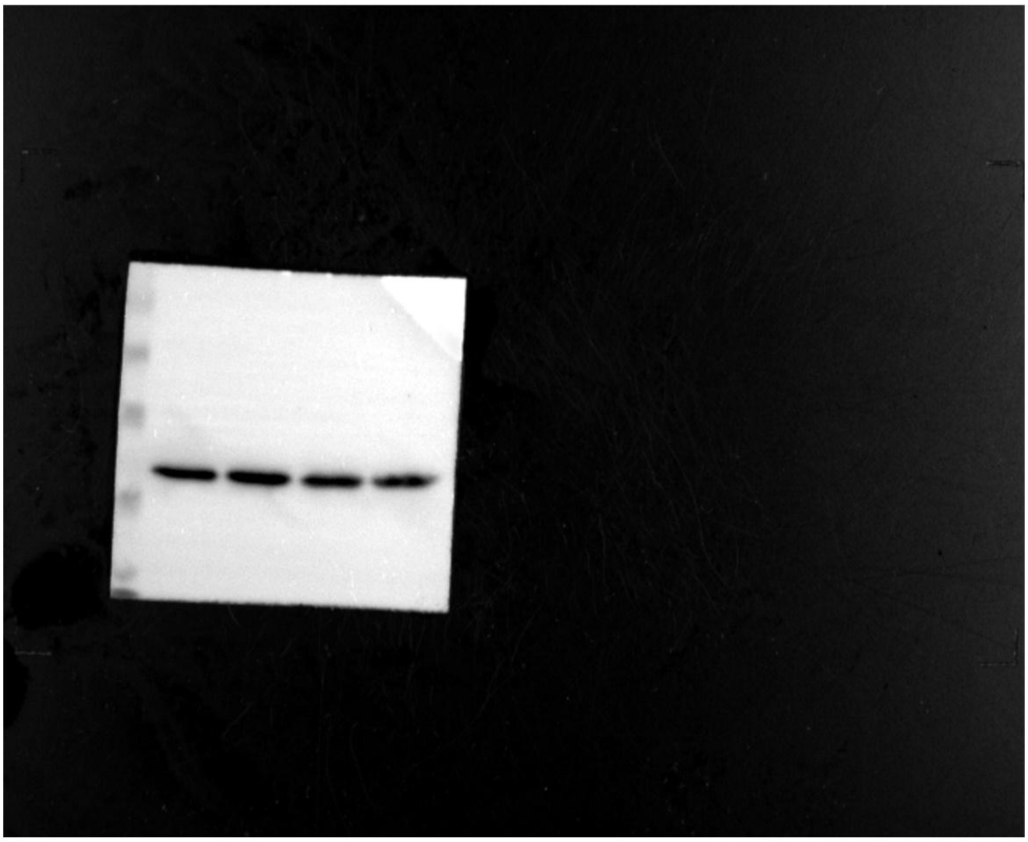

Supplement: S2 File — (PDF) [file pone.0322708.s003.pdf]
